# Host and pathogen DNA identification in blood meals of nymphal *Ixodes ricinus* ticks from forest parks and rural forests of Poland

**DOI:** 10.1007/s10493-013-9763-x

**Published:** 2013-12-19

**Authors:** Beata Wodecka, Anna Rymaszewska, Bogumila Skotarczak

**Affiliations:** Department of Genetics, University of Szczecin, Felczaka 3c, 71-412 Szczecin, Poland

**Keywords:** Blood meal, *Borrelia*, *Anaplasma*, *Rickettsia*, Reservoir, *Ixodes ricinus*

## Abstract

DNA analysis of blood meals from unfed nymphal *Ixodes ricinus* allows for the identification of tick host and tick-borne pathogens in the host species. The recognition of host species for tick larvae and the reservoirs of *Borrelia*, *Rickettsia* and *Anaplasma* species were simultaneously carried out by analysis of the blood meals of 880 questing nymphal *I. ricinus* ticks collected in forest parks of Szczecin city and rural forests in northwestern Poland that are endemic areas for Lyme borreliosis. The results obtained from the study indicate that *I. ricinus* larvae feed not only on small or medium animals but also on large animals and they (i.e. roe deer, red deer and wild boars) were the most prevalent in all study areas as the essential hosts for larvae of *I. ricinus*. The composition of medium and small vertebrates (carnivores, rodents, birds and lizards) provided a more diverse picture depending on study site. The reservoir species that contain the most pathogens are the European roe deer *Capreolus capreolus,* in which two species of *Rickettsia* and two species of *Borrelia* were identified, and *Sus scrofa,* in which one *Rickettsia* and three *Borrelia* species were identified. *Rickettsia helvetica* was the most common pathogen detected, and other included species were the *B. burgdorferi* s.l. group and *B. miyamotoi* related to relapsing fever group. Our results confirmed a general association of *B. garinii* with birds but also suggested that such associations may be less common in the transmission cycle in natural habitats than what was thought previously.

## Introduction

Ticks belonging to the *Ixodidae* family play an important role in the epidemiology of disease transmission to humans and animals. They are also an important element in maintaining the natural outbreaks of these diseases. Development and realisation of control procedures for tick-borne diseases require a detailed understanding of transmission pathways in the wild and, in particular, the identification of new reservoir hosts (Gariepy et al. 2012). Complete knowledge of the host-feeding patterns of tick populations in nature is a critical part of evaluating their vector competence and assessing the role of various vertebrates to serve as reservoir hosts of vector-borne pathogens (Kent 2010; Gariepy et al. 2012). The identification of tick hosts and reservoir hosts is difficult because it requires animal trapping and animal maintenance in the laboratory if xenodiagnosis is required. Host identification from blood meal sucked by a hematophagous vector has significantly facilitated such studies. DNA analysis of blood meal from unfed nymphal *Ixodes ricinus* allows identification of the hosts on which the larvae fed as well as tick-borne pathogens in the host species.

Molecular protocols for detection of host DNA are in constant development for the use of new targets and primers. For the differentiation of reservoirs and *Borrelia* species, Kirstein and Gray (1996) used the mitochondrially encoded *cytb* gene as the molecular target and digested the PCR products using the restriction endonucleases HaeIII and DdeI, a protocol that allowed to distinguish 11 animal species. Pichon et al. (2005) identified vertebrate DNA in the tick gut using PCR amplification with universal primers targeting a portion of the 18S rRNA gene, followed by reverse line blot (RLB) hybridisation. In the study of Humair et al. (2007), a 12S rDNA gene fragment was used for the first time as a PCR marker for detection of host DNA in nymphs.

The present paper is a study concerning molecular identification of *I. ricinus* hosts and *Borrelia* hosts by analysis of the blood meal in ticks in northwestern Poland using PCR–RFLP protocol with 12S rDNA gene fragment as molecular target. This research aims to identify host species of *I. ricinus* larvae, keeping in mind that immature stages of this tick species may feed not only on small animals but have the widest range of host species (L’Hostis et al. 1995; Kiffner et al. 2010). This research also aims to identify the reservoir species for *Anaplasma phagocytophilum*, *Borrelia* and *Rickettsia* and to elucidate any association between *B. burgdorferi* s.l. species and some vertebrate hosts by analysis of DNA from the midgut of blood-feeding nymphal ticks.

## Materials and methods

### Study material and collection sites of *Ixodes ricinus*

The study involved 880 specimens of unfed nymphs of *I. ricinus* collected in four study areas in northwestern Poland (Osow I, Szczecin Landscape Park II, Zielonczyn III and Pobierowo IV). These sites are known to be high risk areas for *Borrelia* infection in ticks (Wodecka 2003). Urban forests constitute 60 % of the city of Szczecin (northwestern Poland) and are mostly composed of forest complexes termed forest parks. The two selected sites, I and II, are the main recreational places for the population of Szczecin throughout the year. Site I is located in the city, Site II is on its outskirts, and large housing estates are situated within both areas. Site I is included in the forest complex of Szczecin, part of which lies at an altitude of 100–130 m above sea level, which provides a upland climate. The rest of the forests within Site I are located in the lowlands and depressions formed in areas rich in vegetation, peat meadows and marshes. In these biotopes, different groups of animals can be found, from invertebrates such as insects and arachnids, which are potential vectors for pathogens, to vertebrates, their potential reservoir. This is also a place for the convergence of bird flight paths in the meridional and latitudinal directions. One of the warmest climatic regions in Poland is Szczecin Landscape Park, which is influenced by the Atlantic climate and is characterised by small fluctuations in the daily and annual temperatures, mild winters, a high amount of rainfall per year and a good distribution of rainfall during the vegetation period. Both of these urban areas, Sites I and II, are characterised by different species of trees with a predominance of pines (*Pinus* spp.), oak (*Quercus* spp.), beech (*Fagus* spp.) and alder (*Alnus* spp.). Among the most common vegetation are fern (*Polypodium vulgare*), stinging nettle (*Urtica dioica*), mosses (*Bryophyta* spp.) and grass (*Poaceae* spp.). The other two tick collection sites were a mixture of forests often visited by strollers and mushroom pickers and farms (with a majority of Scots pine (*Pinus silvestris*), beech (*Fagus sylvatica*) and sessile oak (*Quercus sessilis*) with well-developed forest lining) located approximately 100 km to the west (Site III) and north (Site IV) of Szczecin. Zielonczyn, Site III, is composed of a small village around forest areas and wetlands, as well as pasture land in the Lower Oder Valley, which is located slightly further to the west. Water resources exist in the form of lakes and rivers located in the area, and a large forest area forms a temperate climate characterised by considerable humidity, which can be described as a warm temperate zone. Site IV includes forests near Pobierowo and sea bathing areas in northwestern Poland. The neighbouring marine basin, a pine forest with rich undergrowth, and vast meadows growing on peat contribute to the maintenance of this region-specific microclimate. Mild winters with a long frost-free period, high relative humidity and small temperature fluctuations throughout the year (IMWM 2010) favour the development of wildlife, especially small invertebrates that are involved in the circulation of microorganisms in the environment. Urban and rural forests are the habitat of many species of vertebrates, including large (wild boar, roe deer, red deer), medium and small (fox, raccoon, badger, marten, skunk, muskrat, rabbits, voles, shrew) animals. All sites have been monitored for ticks and tick-borne pathogens by us for several years (Skotarczak et al. 1999, 2008; Rymaszewska 2003; Wodecka 2003; Wodecka and Skotarczak 2005; Wodecka et al. 2010). Ticks were collected by sweeping up the vegetation up to 1 m with a flannel flag and then stored at −20 °C until DNA isolation.

### DNA extraction

DNA extraction from ticks was carried out according to the phenol–chloroform protocol. All tick individuals (nymph or adult) were crushed using a ceramic pestle and suspended in 500 μl of 2× buffer (0.19 M NH_4_Cl, 0.011 M KHCO_3_ and 0.024 M EDTA) with the addition of 100 μl of Lysis buffer (0.017 M SDS, 0.01 M TRIS, 0.01 M EDTA) and 1 μl of Proteinase K (20 mg/ml) (BioShop, Canada). Subsequently, ticks were placed in a 56 °C water bath for 3 h. Following the incubation, 300 μl of phenol (BioShop) was added, and the tube was vortexed for 30 s and centrifuged for 10 min at 9,000 rpm. The supernatant was transferred successively to three additional tubes containing 400 μl of phenol–chloroform (1:1) and 300 μl of chloroform (POCH, Poland) (twice), then vortexed for 30 s and centrifuged for 10 min at 9,000 rpm. Finally, the supernatant was transferred to the last tube, and DNA was precipitated by adding 500 μl of isopropanol (POCH). The pellet was rinsed with 70 % ethanol and air-dried before suspension in Tris–EDTA (TE) buffer (pH 8.0) and the samples were stored at −70 °C until PCR analysis.

### Detection of *Ixodes ricinus* host DNA in blood meal remnants by nested PCR

The mitochondrion-encoded 12S rDNA gene was used as the molecular marker for the detection of tick hosts by nested PCR. Two primer sets were used: outer 532fl2s (5′-CAAACTGGGATTAGATAC-3′) and 1l02rl2 s (5′-TGCTTACCTTGTTACGAC-3′), with a product length of approximately 520 bp, and inner 539f12s (5′-GGATTAGATACCCCACTATGC-3′) and 1015rl2 s (5′-TGAGGAGGGTGACGGGCGGT-3′), with a product length of approximately 440 bp.

The first PCR amplification was performed in a reaction volume of 10 μl containing 0.5 U of Allegro Taq DNA polymerase (Novazym, Poland), 70 mM Tris–HCl (pH 8.6 w 25 °C), 16.6 mM (NH_4_)_2_SO_4_, 3.5 mM MgCl_2_, 0.75 μM of deoxyribonucleoside triphosphate (Novazym), 2 pmol of the two outer primers, 532fl2s and 1l02rl2s, and 1 μl of the supernatant of the processed DNA sample in TE buffer. For the second PCR amplification, 1 μl of a 1:10 dilution of the first PCR product was added to 9 μl of reaction mixture prepared with the inner primers 539f12s and 102r12s. PCR was performed in thermal cyclers T-gradient (Biometria, Germany) and Peltier Thermal Cycler 200 (MJ Research, USA). Templates were subjected to an initial denaturation step of 94 °C for 10 min., followed by 40 cycles consisting of 94 °C for 30 s, 60 °C for 45 s and 72 °C for 1 min. In each PCR run the DNA of eleven vertebrate species were used as positive controls, i.e. *Cervus elaphus*, *Capreolus capreolus*, *Dama dama* and *Sus scrofa* (hunted near Dobra Szczecińska, Zachodniopomorskie province, Skotarczak et al. 2008), *Vulpes vulpes*, *Meles meles* and *Nyctereutes procyonoides* (hunted in Wielkopolska National Park) and *Coccothraustes coccothraustes*, *Sturnus vulgaris*, *Turdus merula* and *Turdus philomelos* (captured in Wielkopolska National Park, Michalik et al. 2008). TE buffer was applied as a negative control. The PCR products were analysed on a 1.5 % agarose gel at 5 V/cm for 1 h. Nova 100 DNA Ladder (Novazym, Poland) was applied for evaluation of the obtained product size. The results of the PCR amplification were viewed under UV light and were archived using BioCapt software (Vilber Lourmat, France).

### Identification of *Ixodes ricinus* hosts by PCR–RFLP

The DNA amplified with primer set 539fl2s and 1015r12s was digested with enzymes AluI, Cfr13I, Tru1I, HpyF31, TaiI and BsuRI. These enzymes were selected on the basis of computer analysis (DNAMAN) of the *12S rDNA* gene sequence downloaded from GenBank that shown there is possibility to obtain RFLP patterns of 60 vertebrate species. Among them were 23 bird species, 5 reptile species and 32 mammalian species (mice, hares, rabbits, bats, hedgehogs, minks, dormice, roe deer, wild boars, red deer, fallow deer)—the species represented in sites selected for the collection of *I. ricinus*.

### Detection of *Borrelia burgdorferi* s.l. DNA by nested PCR–RFLP

A nested PCR method with two primer sets (outer 132f and 905r and inner 220f and 823r) was used to detect the *flaB* gene fragment of *B. burgdorferi* s.l. described earlier (Wodecka et al. 2010; Wodecka 2011). DNA isolated from a reference strain of *B. burgdorferi* s.s. IRS (German Collection of Microorganisms and Cell Cultures—DSMZ, Germany) was used as a positive control and TE buffer was used as a negative control. The PCR products were separated on a 1.5 % agarose gel (Prona, Spain) with the addition of ethidium bromide (Sigma-Aldrich, Germany) at 5 V/cm for 1 h. The MW1444 molecular marker (Polgen, Poland) was applied for evaluation of the size of the obtained product. The results of the PCR were viewed under UV light and were archived using BioCapt software (Vilber Lourmat, France). The DNA of *flaB* gene fragments amplified with primer set 220f and 823r were digested with enzymes HpyF3I and Ecl136II (Fermentas, Lithuania) to obtain the RFLP patterns of different *Borrelia* species, as described elsewhere (Wodecka 2011). The digestion products were analysed on a 3 % agarose gel at 5 V/cm for 2 h and archived as described above.

### Detection of *Anaplasma phagocytophilum* and *Rickettsia* sp. DNA by PCR

The presence of *A*. *phagocytophilum* was detected with a 334 bp-long fragment of the *msp2* gene marked by primers msp2-3F and msp2-3R (Levin et al. 2002). To detect the presence of *Rickettsia* DNA, we used the sequences of the *gltA* gene (citrate synthase), amplified using primers RpCS877 and RpCS1258 (382 bp product) (Nilsson et al. 1999). Phusion High-Fidelity DNA Polymerase (Finnzymes, Finland) was used for the PCR amplification at a mixture concentration 0.5 U/20 μl. Final reagent concentrations were 10 mM Tris–HCl (pH 8.3), 50 mM KCl, 1.5 mM MgCl_2_, 200 μM for each deoxynucleoside triphosphate, 10 pM for each primer and 2 μl DNA. The PCR regime was adjusted to the requirements of Phusion High-Fidelity DNA Polymerase according to the manufacturer’s recommendations. The results of PCR amplification were visualised by electrophoresis of 5–8 μl of each sample in 1.5 % agarose gels with ethidium bromide.

### Contamination control procedures

To minimize contamination, the reagent setup, extraction, sample addition, and the PCR and sample analyses, were performed in 3 separate laboratories. Additionally, validation of restriction patterns for nested PCR products was performed by sequencing the amplicons obtained with primers 539f12s and 102r12s. Sequencing was performed for each positive control sample and study sample being representative of each restriction pattern for different vertebrate species. The 12S rRNA sequences of 32 strains determined in this study were deposited in GenBank under the accession numbers listed as follows: KF781309 (*Accipiter gentilis*), KF781310 and KF781334 (*Capreolus capreolus*), KF781311 (*Castor fiber*), KF781312 (*Ciconia nigra*), KF781313 and KF781335 (*Cervus elaphus*), KF781314 and KF781336 (*Dama dama*), KF781315 (*Lacerta viridis*), KF781316 (*Lepus europaeus*), KF781317 (*Myodes glareolus*), KF781318 and KF781338 (*Meles meles*), KF781319 (*Natrix natrix*), KF781320 and KF781321 (*Oryctolagus cuniculus*), KF781322 (*Perdix perdix*), KF781323 and KF781337 (*Sus scrofa*), KF781324, KF781325 and KF781330 (*Turdus merula*), KF781326 and KF781331 (*Turdus philomelos*), KF781327 (*Upupa epops*), KF781328 and KF781339 (*Vulpes vulpes*), KF781329 (*Zootoca vivipara*), KF781332 (*Coccothraustes coccothraustes*), KF781333 (*Sturnus vulgaris*), KF781340 (*Nyctereutes procyonoides*).

## Results

### Hosts DNA

DNA of potential *I. ricinus* larvae hosts were detected in 553 out of 880 nymphs (62.8 %). We obtained 19 types of restriction patterns consistent with the analysis of the *12S rRNA* gene sequence. In total, 19 species of animals were identified: 10 mammals, 6 birds and 3 reptiles (Table 1). In each collection site the main hosts for *I. ricinus* larvae were large mammals, including wild boars, red deer and roe deer (Table 1). They constituted from 54.8 to 60 % of samples with detected host DNA and differences between every pair of compared sites were not statistically significant (*p* > 0.624). The differences between the same identified species were statistically significant in case of red deer when two forest parks were compared (*p* = 0.001) and in case of wild boars between each compared pair of sites (*p* < 0.049) excluding two rural forests comparison. The second most prevalent group of tick hosts in forest parks were birds and their participation ranged from 34.1 to 35.2 % of samples with detected host DNA in contrary to rural forests (5.5 and 6.8 %, Table 1). The differences were statistically significant when both types of habitats were compared (*p* < 0.001). Similar results were obtained in case of medium and small mammals that were the second most prevalent group of detected tick hosts in rural forest. In mentioned habitat they were identified in 29.5 % (Pobierowo) and in 30.8 % (Zielonczyn) whereas in forest parks their prevalence ranged from 4.8 to 7.5 % (Table 1). The differences were statistically significant when different habitats were compared (*p* < 0.001).

**Table 1:** Identification of host origin of the blood meal remnants in nymphs of *Ixodes ricinus* collected from forest parks and rural forests

Host species	Number of *I. ricinus* nymphs with the host DNA (n/%)			
	Forest parks		Rural forests	
	Osow (I) (105) n/%	Szczecin Landscape Park (II) (161) n/%	Zielonczyn (III) (104) n/%	Pobierowo (IV) (183) n/%
Large mammals				
*Cervus elaphus* (red deer)	10/9.5	51/31.7	19/18.3	33/18.0
*Capreolus capreolus* (roe deer)	20/19.0	29/18.0	20/19.2	36/19.7
*Dama dama* (fallow deer)	–	4/2.5	–	3/1.6
*Sus scrofa* (wild boar)	33/31.5	8/5.0	18/17.3	32/17.5
Total	63/60.0	92/57.2	57/54.8	104/56.8
Medium and small mammals				
*Vulpes vulpes* (red fox)	–	–	15/14.4	37/20.2
*Meles meles* (European badger)	5/4.8	–	2/1.9	–
*Castor fiber* (European beaver)	–	–	1/1.0	–
*Lepus europaeus* (European hare)	–	7/4.4	–	–
*Oryctolagus cuniculus* (European rabbit)	–	5/3.1	4/3.9	–
*Myodes glareolus* (bank wole)	–	–	10/9.6	17/9.3
Total	5/4.8	12/7.5	32/30.8	54/29.5
Birds				
*Accipiter gentilis* (northern goshawk)	1/1.0	2/1.2	–	–
*Ciconia nigra* (black stork)	–	–	1/1.0	–
*Perdix perdix* (grey partridge)	15/14.3	13/8.1	–	–
*Turdus philomelos* (song thrush)	8/7.6	15/9.3	2/1.9	2/1.1
*Turdus merula* (common blackbird)	13/12.3	24/14.9	4/3.9	8/4.4
*Upupa epops* (hoopoe)	–	1/0.6	–	–
Total	37/35.2	55/34.1	7/6.8	10/5.5
Reptilians				
*Lacerta viridis* (green lizard)	–	–	7/6.6	15/8.2
*Zootoca vivipara* (viviparous lizard)	–	2/1.2	–	–
*Natrix natrix* (grass snake)	–	–	1/1.0	–
Total	–	2/1.2	8/7.6	15/8.2
Host DNA not detected	56	93	47	131
Number of tested nymphs	161	254	151	314

### Pathogens’ DNA

DNA of tick-borne pathogens was detected using nested PCR protocol in 97 of 880 nymphs (11 %). The vast majority of pathogens detected in the nymphs were *Rickettsia* species (71.1 %, Table 2). The DNA of *A. phagocytophilum* was not detected in any ticks, but the DNA of *Borrelia* was affirmed in 27.8 % of all infected nymphs. *B. garini* and *B. afzelii* from the *B. burgdorferi* s.l. group were more frequent than *B. miyamotoi* of the relapsing fever group. Double infection of *B. garinii* and *R. helvetica* was observed once (Table 2).

**Table 2:** Correlations of pathogens with hosts based on DNA detection in *Ixodes ricinus* nymphs (98 pathogens in 97 ticks)

Host DNA	*Sus scrofa*	*Capreolus capreolus*	*Lacerta vivipara*	*Meles meles*	*Perdix perdix*	*Cervus elaphus*	*Myodes glareolus*	*Oryctolagus cuniculus*	*Turdus merula*	Host DNA not detected	Total n/%
Pathogen											
*Rickettsia helvetica*	7	15	1	–	1	7	1	–	–	37	69/71.1
*Rickettsia monacensis*	–	1	–	–	–	–	–	–	–	–	1/1.03
*Anaplasma phagocytophilum*	–	–	–	–	–	–	–	–	–	–	0/0
*Borrelia afzelii*	1	1	–	1	–	1	–	1	–	4	9/9.24
*Borrelia garinii*	2	–	–	1	1	–	1	–	2	3	10/10.3
*Borrelia lusitaniae*	–	–	–	–	–	–	–	–	–	1	1/1.03
*Borrelia miyamotoi*	2	2	–	–	–	–	–	–	1	1	6/6.2
*B. garinii/R. helvetica*	–	–	–	–	–	–	–	–	–	1	1/1.03
Total	12	19	1	2	2	8	2	1	3	47	97/100

### Correlation of pathogens and hosts

The records of pathogen occurrence with respect to host DNA demonstrated that *R. helvetica* was mainly associated with *Capreolus capreolus*, *Sus scrofa* and *Ce. elaphus*, but the blood meal origin could not be determined in 37 ticks infected with this species (Table 2). DNA of *B. afzelii* was found in ticks that had fed on five species of animals, including large mammals (red deer, roe deer and wild boar) and medium ones (badger and rabbit). *B. garinii* also correlated with five host species, and *B. miyamotoi* with three host species (Table 2). The reservoir species that contained the most pathogens was the European roe deer *C. capreolus,* in which two species of *Rickettcia* and two species of *Borrelia* were identified: one from the *B. burgdorferi* s.l. group (*B. afzelii*) and one from the relapsing fever group, *B. miyamotoi.*

## Discussion

The protocol applied in our study (PCR–RFLP) allows to detect 60 vertebrate species, including 32 mammalian species, 23 bird species, and five reptile species that are strictly connected with forest habitat and therefore may serve as hosts for ticks, especially for common tick *I. ricinus*. The DNA of potential hosts of *I. ricinus* larvae was detected in 553 nymphs (62.8 %). We obtained 19 types of restriction patterns consistent with those predicted on the basis of *12S rRNA* gene sequences derived from GenBank.

Ticks were collected across a range of habitat types: two sites were chosen in the city and two in the rural forests. In each site the main hosts for *I. ricinus* larvae constitute large mammals (54.8–60 %) including roe deer, red deer and wild boars. Despite of accordance in the participation level of large mammals as tick hosts individual mammal species demonstrated site specific distribution: red deer were most prevalent in one forest park (Szczecin Landscape Park) and wild boars in the second (Osów) whereas in rural forests there were equal distributions of three mentioned species.

In the forest biotope of Szczecin (I-II), the crucial hosts for *I. ricinus* larvae, apart from large mammals, were birds. Sites I and II are frequently visited by humans, who are attacked but not constitute as hosts for *I. ricinus* larvae, nymphs and adults to maintain their population. Sites I and II are often exploited by dog owners, walkers, joggers and cyclists, which constrain undergrowth (mostly grasses, mosses, bilberries and ferns), such that only small game may inhabit these sites. In this case human rather disturb the vegetation scaring away small vertebrate fauna which may explain the lack of host rodents for larvae. Our study revealed that in these sites, this gap is filled by birds. In the contrary, the results of Estrada-Pena et al. (2005) showed a strong infestation of birds with the simultaneous presence of rodents in Spain. Pichon et al. (2005) found that songbirds are the most significant hosts in a forest on the outskirts of Berlin (Germany). In both above studies the results were obtained for the gene target located in the nucleus and for this reason the method would have discriminated in favour of birds comparing to our results.

In the two selected rural forest sites (III-IV), large mammals are also major hosts, though medium and small mammals and lizards also play significant roles. The results obtained in this and other studies suggest that the assumption that the immature stages of *I. ricinus* feed on small- and medium-sized mammals and that imago feed on large mammals (Kurtenbach et al. 1995; Liz et al. 2000; Hanincová et al. 2003a; Rizzoli et al. 2004; Michalik et al. 2005) should be verified. Thus, all stages of *I. ricinus* feed on roe deer (Kiffner et al. 2010), red deer and wild boars. It is known that the abundance of *I. ricinus* ticks is affected by habitat structure, climate and host community composition, but the claims by Jackson et al. (2006), Paziewska et al. (2010) and others—that changes in land use and greater human penetration of forest habitats are likely to have a greater force on the occurrence of tick-borne disease than climate changes—are well founded.

In the present study, the DNA of pathogens was detected in 97 out of 880 nymphs (11 %). The DNA of *Borrelia* was in 27.8 % of all infected nymphs and corresponded to 3 species of *B. burgdorferi* s.l. and one species of relapsing fever related *B. miyamotoi*. A double infection involving *B. garinii* and *R. helvetica* was observed once. Data on the host specificity of *B. burgdorferi* s.l. species (Gray et al. 2000; Kurtenbach et al. 2002; Hanincová et al. 2003b) suggest that *B. valaisiana* and *B. garinii* are mostly associated with birds, and the high proportion of these species at these sites points to birds as the main reservoir hosts of the species complex. The DNA analysis of tick hosts detected in blood meal remnants of *I. ricinus* nymphs in several contexts by Wodecka (2008) (*Turdus merula, T. philomelos* and *Phasianus colchicus*) and in the present study (*Perdix perdix* and *Accipiter gentiles*) confirms the conclusion above. Nevertheless, the DNA of *B. garinii* was detected alongside the DNA of *Sus scrofa*, *Meles meles* and *Pedrix pedrix* as well. The DNA of *B. afzelii* was found in ticks that had fed on 5 species of large and medium mammals, even though according to several authors, this species of spirochete is associated with rodents. Our results and those of other authors (Estrada-Pena et al. 2005; Pichon et al. 2006; Franke et al. 2010) seem to suggest that loose associations exist in the transmission cycle of *Borrelia* species in nature in various geographical areas. The DNA of *B. miyamotoi* was found in ticks that had fed on 4 species of animal hosts, including large mammals and hawks. *B. lusitanie* DNA was detected in the Polish population of *I. ricinus* for the first time in 2005 (Wodecka and Skotarczak 2005), and in the current study, DNA of host for this species was not identified.

The animal species in which the most *Borrelia* species occurred was *Sus scrofa*. The most pathogens were found in the European roe deer *C. capreolus,* in which two species of the *Borrelia* genus were identified, one from the *B. burgdorferi* s.l. group (*B. afzelii*) and one species of relapsing fever *B. miyamotoi,* as well as two species of *Rickettsia*.

Based on an analysis of unfed *I. ricinus* nymphs, Estrada-Pena et al. (2005) found that wild boar may be a reservoir species for *B. afzelii*. However, most of the evidence, especially that obtained from field studies, indicates a lack of reservoir competence of ungulate animals (European Union Concerted Action on Lyme Borreliosis—EUCALB). Generally, the ungulate animals play a large role in the epidemiology of borreliosis and the circulation of spirochetes in nature due to their role as hosts to *I. ricinus* ticks, which can be infected with pathogens through co-feeding with other ticks (Ogden et al. 1997; Juricová and Hubálek 2009). Thus far, it has been found that large forest mammals, such as wild boars, roe deer, red deer and those that are basic hosts for adult *I. ricinus,* are not able to constitute a reservoir for the species belonging to *B. burgdorferi* s.l. complex. Currently, many factors that determine a host’s sensitivity or resistance to *Borrelia* are known, but the crucial factor that could explain the selective transmission and host association of *B. burgdorferi* s.l. is the lytic component in serum, which was identified as the alternative pathway of complement (Kurtenbach et al. 2002). Analyses of resistance or sensitivity patterns to complement have been extended to many *B. burgdorferi* s.l. strains and different vertebrate species.

Perkins et al. (2006) demonstrated that animals such as deer are important blood hosts for feeding *I. ricinus* ticks but that they do not support transmission of many tick-borne pathogens, instead acting as dead-end transmission hosts. Deer maintain high tick intensities, which perpetuate tick populations, but they do not support tick-borne pathogen transmission, so they are dilution hosts. In our earlier molecular studies (Skotarczak et al. 2008) elucidating the role of game in the circulation of pathogens transmitted by ticks in northwestern Poland, DNA was isolated from tissues (blood and spleen) of roe deer, red deer and wild boars. The results showed that *C. capreolus* and *Ce. elaphus* play an important role as reservoirs of *A. phagocytophilum*, two *Bartonella* species, *Babesia divergens* and *Theileria* sp. but not that of *Borrelia.* Whereas the DNA of only one pathogen, *A. phagocytophilum,* occurred in the isolates obtained from 50 representatives of *S. scrofa*, in the present study, the DNA of *A. phagocytophilum* was not detected in any tick. However, that result is not surprising, as the presence of this pathogen is maintained at a relatively low level in north-western Poland (Rymaszewska 2004; Skotarczak et al. 2006, 2008).

The highest percentage of pathogens detected in the nymphs was the *Rickettsia* species (72 %). The results on pathogen occurrence in relation to DNA in blood meal demonstrated that *R. helvetica* was mainly associated with *C. capreolus*, *S. scrofa* and *Ce. elaphus*, but the blood meal origin could not be determined in 37 ticks infected with this bacterial species. *R. helvetica* is very widespread in Europe and has been detected in *I. ricinus* in several countries, including Italy (Beninati et al. 2002), Spain (Fernandez-Soto et al. 2004), Southern Germany (Hartelt et al. 2004) and Denmark and Sweden (Nilsson et al. 1999; Nielsen et al. 2004). The second species whose DNA we found together with roe deer DNA in engorged nymphal *I. ricinus* was *R. monacensis,* which is very rare in tick collection sites in north-western Poland (Rymaszewska and Piotrowski 2013) but is more often detected in *I. ricinus* in other countries, e.g. Germany (Schorn et al. 2011). *R. monacensis* was identified as an etiologic agent of a Mediterranean spotted fever (MSF)-like illness in Spain (Jado et al. 2007). Madeddu et al. (2012) reported a case of MSF-like illness in a 28-year-old man from Sardinia. *I. ricinus* ticks are considered to be vectors of *R. monacensis* and have been found in Sardinia, although less often than other tick species. However, other ticks might act as vectors for *R. monacensis* in Sardinia, where ticks of the genus *Rhipicephalus* are prominent. The identification of *R. monacensis* as a cause of an MSF-like illness expands the list of pathogenic rickettsiae circulating in Italy.

Known species of *Rickettsia* are the only tick-borne pathogens transmitted transovarially with 100 % efficiency. Generally, tick-transmitted *Rickettsia* species persist for a short time in vertebrate organisms, making the bacteremia difficult to detect (Raoult and Roux 1997). Nevertheless, recent studies indicate that some vertebrate animals may be a reservoir for *Rickettsia*. De Sousa et al. (2012) indicated a potential role of the *Teira dugesii* lizard species in the maintenance and transmission cycle of *I. ricinus* tick-borne agents such as *R. monacensis* and *R. helvetica* that are circulating on Madeira Island, and Franke et al. (2010) considered the reservoir role of birds on a conservation island in the Baltic Sea. Additionally, our study (unpublished data) revealed the presence of *R. helvetica* DNA in the blood of a goat *Capra hircus* (9 %). In Japan, two independent teams confirmed the presence of *R. helvetica* DNA in the blood of sika deer (*Cervus nippon yesoensis*) and in raccoons (*Procyon lotor*) (7.1 and 1.6 %, respectively) (Inokuma et al. 2008; Sashika et al. 2010). In addition, Seino et al. (2008) detected a related species, *R. asiatica,* in the blood of *C*. *nippon yesoensis* in Hokkaido.

The presented research results show that the nymphal *I. ricinus* ticks, which parasitize deer in the larval stage, were most often infected by *Rickettsia*. A relatively high level of infection may indicate that the infection is not coincidental and that these results are not simply a consequence of the transovarial transfer of rickettsiae. Therefore, the hypothesis about the role of ruminants in the circulation of bacteria of the genus *Rickettsia* seems probable and should be confirmed in further studies.
